# The Physiology Associated With “Bed Rest” and Inactivity and How It May Relate to the Veterinary Patient With Spinal Cord Injury and Physical Rehabilitation

**DOI:** 10.3389/fvets.2021.601914

**Published:** 2021-03-12

**Authors:** Stephanie A. Thomovsky

**Affiliations:** Department of Veterinary Clinical Sciences, Purdue University, West Lafayette, IN, United States

**Keywords:** neurology, neurosurgery, bed rest, cage rest, physical rehabilitation, post-operative, spinal cord, myelopathy

## Abstract

In the twentieth century, bed rest was commonly prescribed by human healthcare professionals as a treatment for a variety of ailments including spinal cord injury and disease. With time, the negative impact of bed rest was recognized as a source of slow and even reduced patient healing. As treatment paradigms shifted, the utility and importance of physical rehabilitation (PR) as a critical adjunctive treatment for human patients with spinal cord injury became fully recognized. Today, standardized PR protocols exist for humans with the spinal cord disease, but the same cannot be said for our veterinary patients with spinal cord injury. The purpose of this manuscript is to discuss the effects of inactivity on the musculoskeletal system and to explore how and why PR can play a critical role in improved mobility and overall health in the veterinary patient with spinal cord injury. Research with a focus on the effects of inactivity, in the form of cage rest, for the veterinary patient with spinal cord injury is lacking.

## Introduction

Bed rest has long been a component of healing and often a prescribed treatment for various ailments in human medicine. The rationale for bed rest as a treatment is to reduce the metabolic demand of the body, thus improving healing and recovery. The regular use of bed rest as a treatment dates back to the nineteenth century (1, 2). The utility of bed rest was not challenged until the Second World War when out of a necessity for hospital bed space, soldiers were encouraged to rise and become mobile at a faster rate following an injury. The same individuals were noted to recover from their injuries and infections at quicker rates than soldiers who were confined to the bed for longer periods of time (2, 3). In the 1960s, human spaceflight research revealed parallels between the bodily changes observed in humans living in environments lacking gravity and patients who are bedridden. This research further moved doctors away from bed rest as a medical prescription for healing (2). The deleterious effects of inactivity on the musculoskeletal, nervous, cardiopulmonary, hematologic, gastrointestinal, endocrine, renal, and immune systems have been well-established in humans (1–5). Inactivity also can affect the psychological state and mental health of a patient (1, 2, 4, 5).

One could argue that the veterinary equivalent to bed rest is cage rest. Strict cage rest is commonly used in veterinary medicine as a standard of treatment for dogs with spinal cord injury and disease. A recent study found that 87% of veterinary neurosurgeons surveyed recommend 2–8 weeks of strict cage rest to their patients following spinal surgery (6). The important purpose of this rest cannot be denied in a species that is unable to grasp the concept of reinjury and neurologic worsening secondary to over activity; cage rest allows time for the spinal column to stabilize. That being said, the deleterious effects of cage rest must also be considered.

One treatment used to combat the deleterious effects of bed rest and inactivity is controlled physical rehabilitation (PR). PR has been shown, in humans, to successfully improve postoperative recovery rates, reducing pain, improving mobility, range of motion, balance, cardiovascular fitness, and patient mental health (7). Today, specifically with respect to human patients with spinal cord injury, there is increasing evidence that exercise can improve healing (8). How exercise, in the form of controlled physical rehabilitation, can be incorporated into treatment in veterinary patients suffering from spinal cord injury and disease is yet to be established. To the author's knowledge, accepted, standardized PR treatment protocols in veterinary patients with spinal cord injury are lacking. For some, PR means passive range of motion and supported 5-min bathroom break walks, and for others it means supported standing, weight shifting, controlled supported leash walks, hydrotherapy, and even electrical stimulation (9). The purpose of this manuscript is to discuss the effects of inactivity in the form of bed rest on the musculoskeletal system and to explore how PR can play a critical role in improved mobility and the overall health in veterinary patients with spinal cord injury.

### Inactivity and the Muscle

Decreased mobility results in disuse muscle atrophy, namely, measured as reduction in the cross-sectional area of unused musculature (10). Muscle loss can be extreme and fast during periods of recumbency. Unused muscle loses strength and atrophies at a rate of 12% per week in humans (4). Studies have shown a 15% loss in the muscle following 1 week of bed rest, increasing to nearly 50% loss after 3–5 weeks of bed rest (4, 11, 12).

There are controversial data on the type of muscle preferentially affected by muscle loss in immobile humans. Some data indicate that type I muscle fibers, i.e., those found in postural muscles, are preferentially affected by an abrupt change in gravity (13). Type I muscle fibers, specifically located in the distal limb, are affected because these muscles are closer to the surface of the earth and, therefore, resist a greater percentage of the gravitational pull of the earth compared to other muscles when a human is standing (1). The calf and soleus muscles are most profoundly affected (14). In the quadruped, distal limb musculature and postural muscles are similarly more profoundly affected by inactivity than other skeletal muscles (13).

Meanwhile, other sources found the strength of contraction in the more metabolically active type II muscle fibers to be more profoundly affected by bed rest and inactivity (1). Yet another article, however, showed no significant difference in the percentage loss of type I vs. II muscle fibers lost with rest (15). Although atrophy was present, the authors stated that the observed decreased muscular strength could not be solely contributed to atrophy. The authors conjectured that reduced force-generating capacity of the muscle in the form of reduced tension and/or reduced or altered peripheral nerve stimulation could be the source of reduced strength in these patients (15).

Corcoran, in 1991, found that during periods of rest, muscles exerting <20% of their normal function would atrophy. In addition to atrophy, contraction and eventual fibrosis are natural sequelae to reduced mobility. This contraction can further potentiate muscle size reduction and atrophy. As the muscle shortens, a reduction in maximal voluntary isometric contraction is observed (10). In addition to contraction, a histopathologic analysis of the inactive muscle reveals a reduction in the number of sarcomeres present (4). The end result of all of these muscle changes is a reduction in the metabolic activity of muscle, in the circulation required for its function, and ultimately loss of muscle strength. This loss of strength is often greater than the degree of disuse atrophy observed (4). Muscle atrophy also leads to alterations in the innervation of the affected muscle; motor unit recruitment is reduced and coordination is affected. These changes in the innervation further contribute to reduced muscle contraction efficiency and clinical weakness (4).

### Inactivity and the Connective Tissues

Inactivity and bed rest also lead to significant changes to connective tissues. Ligament, tendon, and joint capsule contracture are observed, as collagen textures and consistency are altered (4). These contractures begin as early as 8 h after the onset of reduced mobility and can be a cause of stiffness and muscle soreness (11). During periods of inactivity, contracture of the joint occurs because of a combination of joint capsular shortening, adhesive capsulitis, and fibrosis (16). Immobility also leads to alterations within the synovial tissue. Decreased synoviocyte proliferation leads to decreased synovial fluid production. Collagen fibers become disorganized while an increased number of type I collagen fibers are observed (16–19).

Further studies have shown changes to the mechanical properties of ligaments and tendons following periods of inactivity and immobility. Most notable are changes to fibroblasts and collagen fibers. A major role of the fibroblast is to adapt to the changes in mechanical stress in the ligament and to allow for effective motion. Changes to fibroblast morphology and alterations in organelles contained within the fibroblast lead to reduced fibroblast function in as early as a 9-week immobilization period (20). Increases in the synthesis and destruction of collagen fibers result in abnormal and ineffective cross-linking and incorrect fiber orientation—the outcome is reduced ligament and tendon strength (20). Changes observed in the ligaments and tendons following disuse or immobility are time and dose dependent. The longer the tissue is left unused and less stress placed on the tissue, the greater the degree and speed of changes observed within the tendon or ligament (20, 21).

In addition to the tendon, ligament, and joint capsule, the intervertebral disk itself is also altered in shape and structure during periods of inactivity. Humans suffering lower back pain following a 60-day period of head-down tilt bed rest showed increased posterior disk height and multifidus muscle atrophy. These changes in disk dimension in combination with muscle atrophy were thought to be the direct cause of bed rest-induced back pain in the individuals studied (22).

### Inactivity and the Bone

Bone is also altered by inactivity. Gravitational compression, muscle contraction, and the stresses associated with weight-bearing are integral to appropriate bone formation and mineralization (23). In humans, the most significant changes to the bones following inactivity are seen in weight-bearing bones such as those of the vertebral column. The bone mineral density decreased by 1% per week of bed rest (4). This disuse osteoporosis is a sequela to osteoclast cells remaining active while osteoblast cell activity is reduced (4, 10, 23). This form of osteoporosis is quick in onset; the urinary excretion of calcium secondary to bone loss can be observed immediately following the onset of bed rest (11). In addition to disuse osteoporosis, the histologic makeup of bone is altered by inactivity. The intracortical layers of the bone become more porous, the cross-sectional area of the cortical bone is reduced, and increases in endosteal diameter are observed (23). Overall, the bone integrity is significantly reduced during periods of inactivity.

## Role of PR to Improve Mobility Following a Spinal Cord Injury

Physical rehabilitation in patients healing from spinal cord injury can be used to improve mobility and functional weight bearing. The majority of publications exploring the impact of rehabilitation on the speed and efficacy of recovery in canine spinal cord injury have shown improved neurologic function with PR (24–27). Fewer reports, however, have shown no improvement in patients with spinal cord injury having received PR (9, 28). The short- and long-term effects on muscle, connective tissues, bone, and joints following rehabilitation are yet to be studied in these patients. It would stand to reason that exercises, such as range of motion, would be beneficial to maintain and improve muscle and joint elasticity, reducing the likelihood of connective tissue, joint capsule, and muscle contracture. However, standing exercises help improve gravitational compression and basic mechanical stresses with the end result being improved bone loading. Activities, such as walking with or without the use of assisted devices, improve active joint range of motion, muscle, and connective tissue contraction and also encourage normal body position and stance. Walking exercises also activate the use of core and supportive musculature and improve bone loading and cardiopulmonary fitness. Positive effects are observed in the hematologic, gastrointestinal, and psychological systems, as well.

## Conclusions

Inactivity and bed rest have deleterious effects on the body. Changes discussed within the scope of this paper include those observed in the musculoskeletal system, but a variety of changes are also observed in the cardiopulmonary, hematologic, gastrointestinal, endocrine, renal, immune, and nervous systems (1–5). For many veterinary patients with spinal cord injury, inactivity and bed rest in the form of cage rest is a reality. PR can be beneficial to help reverse or decrease the musculoskeletal changes observed with inactivity. Exercises focusing on passive and active range of motion, coordination work, and weight bearing in combination with periods of rest allow appropriate healing and lessen the rapidity of musculoskeletal atrophy in these patients. Future studies focused on the specific effects of cage rest on veterinary patients with spinal cord injury are imperative. Focus can be given to reducing or preventing the negative sequelae to inactivity through the use of PR exercises. Perhaps, in time, effective standardized rehabilitation protocols can be established for these patients.

## Author Contributions

The author confirms being the sole contributor of this work and has approved it for publication.

## Conflict of Interest

The author declares that the research was conducted in the absence of any commercial or financial relationships that could be construed as a potential conflict of interest.
